# Investigation of the Effects of Polyurethane-Modified Polycarboxylate at Ambient Temperature on the Characteristics of Cement with Supplementary Cementitious Materials

**DOI:** 10.3390/polym15173602

**Published:** 2023-08-30

**Authors:** Shuncheng Xiang, Tingxiang Zheng, Jiake Zhang, Zhen Jiang, Bin Liu, Liangjun Huang

**Affiliations:** 1 School of Traffic and Transportation Engineering, Changsha University of Science and Technology, Changsha 410114, China; zzztingx@126.com; 2 College of Transportation Engineering, Tongji University, Shanghai 200092, China; zhjiake@tongji.edu.cn; 3 China West Construction Group Co., Ltd. Hunan Branch, Changsha 410082, China; jianzhen1143@163.com (Z.J.); liubin4010@163.com (B.L.); 4 Huaihua Dongxing Concrete Co., Ltd., Huaihua 418000, China; huang0408tx@yeah.net

**Keywords:** polycarboxylate, supplementary cementitious materials, surface tension, flowability, zeta potential

## Abstract

Via radical polymerization, three polyurethane-modified polycarboxylate molecules of various comb topologies were synthesized. This study investigated the effects of varying types and concentrations of supplementary cementitious materials (SCMs) on the surface tension, flowability, and zeta potential of cement. An elevation in the molar ratio between isoamyl alcohol polyoxyethylene (TPEG) and acrylic acid (AA) from 1:1 to 5:1 reduced the surface tension of the polycarboxylate molecule from 47.70 mN/m to 35.53 mN/m and increased flowability from 280 mm to 310 mm, as the results indicated. An increase in the SCM and polycarboxylate dosage proportionally decreased liquid-phase surface tension and increased flowability. A decrease in the water-to-cement (w/c) ratio from 0.5 to 0.3 corresponded to an observed increase in the zeta potential of cement pastes. However, a rise in the quantity of polycarboxylate and SCMs corresponded to a decrease in the zeta potential at a w/c ratio of 0.3.

## 1. Introduction

Polycarboxylate superplasticizer molecules contain many functional groups, including –COOH, –SO_3_H, –NH_2,_ and –OH groups. The hydration products of Portland cement allow these groups to attach to the products’ surface, which also breaks down the flocculating structure between silicate particles while forming an adsorption layer. The steric hindrance and electrostatic repulsion of the polycarboxylate superplasticizer, when added, can significantly alter the interaction between cement particles at the interface between water and solid activator; this will improve flowability and other chemical properties once the Portland cement particles are evenly distributed [1,2,3,4]. Cement paste is a typical solid–liquid dispersion system, and the solid–liquid interface energy is a key parameter in determining the stability of the system. The addition of polycarboxylate superplasticizer can greatly reduce interfacial energy and improve the dispersion of cement particles. SCMs are necessary components in the preparation of modern concrete. Common SCMs include mineral powder (which refers to ground granulated blast furnace slag powder), fly ash, and metakaolin, among others, which all significantly influence concrete performance [5]. Different water-reducing agents produce different adsorption characteristics when used in Portland cement and SCMs [6,7,8], and such differences make it extremely important to choose suitable water-reducing agents on different occasions. He Yan et al. [9] believed that as polycarboxylate (PCE) molecules become more sulfonated, cement pastes will disperse better. He Yi et al. [10] thought that the higher carboxyl group content of PCE molecules would facilitate the dispersion of cement particles. Chu Hongqiang et al. [11] found that SO_4_^2−^ would increase the dissolution concentration of Ca^2+^ in calcium-silicate-hydrate gel (C–S–H), which increased polycarboxylate superplasticizer aggregates and sharply reduced PCE molecule dispersion in Portland cement. Therefore, it is necessary to strictly control the content of PCE in a concrete system. The research results from Schmid et al. [7] showed that when the polycarboxylate superplasticizer is used, it reduces the flowability of the slurry. When its dispersion of the montmorillonite and kaolin were mixed, the effect of montmorillonite on the dispersion of the system was greater.

Wen, Xiaodong, et al. [12] studied the effect of water-reducing agents on the rheological properties of cement pastes by adjusting the length and density of the side chain in plasticizer molecules and thus obtained the rheological law of the molecular structure of water-reducing agents and their corresponding cement pastes. In a study conducted by Tang Xinde et al. [13], acrylamide (AM), N, N-dimethyl acrylamide (DMAA), and 2-acrylamide-2-methyl propane sulfonic acid (AMPS) were used as functional monomers, and small molecule monomer acrylic acid (AA) was substituted with different molar ratios to study the adsorption capacity of polycarboxylate superplasticizers on the surface of Portland cement particles, based on the free radical polymerization method. Research results showed that the less the negative charge was in water-reducing agent molecules, the lower the degree of condensation. Javadi et al. [14] synthesized a series of water-reducing agents with different side chain densities and studied their effects on the dispersion of Portland cement particles. They found that when the side chain density was between 20% and 25%, both the dispersion and the flowability hold facility of cement particles could be balanced.

From the references above, however, we found that there are few studies on the adsorption and dispersion characteristics of Portland cement and SCMs in combination with different water-reducing agents. In this study, polyurethane-modified polycarboxylate superplasticizer (M-PCE, M-PCE was M for short) with the same main chain length and different side chain lengths was synthesized by controlling the molar ratio of large monomers to methacrylic acid. The interaction between MPCE and Portland cement mixed with supplementary cementitious materials was studied. We hope to accurately regulate the molecular structure of polycarboxylic acids based on polyurethane side chain macromers, effectively elucidate the structure–activity relationship, and reveal the interaction mechanism between polyurethane side chain macromers and Portland cement. The relevant research results are expected to provide sufficient theoretical basis for the design, synthesis, structure–activity relationship, and interaction mechanism with Portland cement of polyurethane polycarboxylate.

## 2. Experiment

### 2.1. Raw Materials

**Figure 1:**
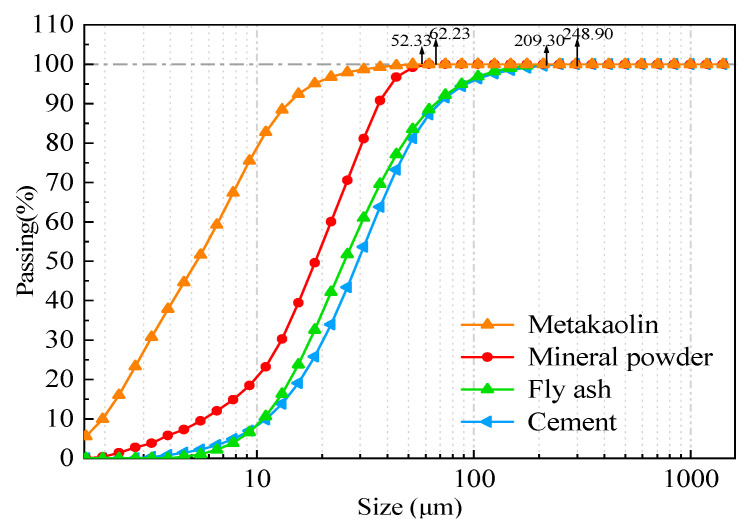
A comparison of cement, mineral powder, fly ash, and metakaolin sizes.

The raw materials included P·I42.5 cement, mineral powder, fly ash, and metakaolin. Their chemical components and particle size distribution are shown in Figure 1 and Table 1. Among them, the particle size distribution of metakaolin, mineral powder, fly ash, and P·I42.5 cement were 6.44 μm, 20.30 μm, 33.69 μm, and 36.96 μm, respectively. 

The raw materials were terminal hydroxy siloxane, dimethyl propionic acid, polyether diethyl alcohol, 1,4-butanediol, isoamyl polyoxyethylene ether (TPEG-2400 g/mol), n-methyl pyrrolidone, and isophorone diisocyanate. Acrylic acid, p-toluenesulfonic acid, thioglycolic acid, ascorbic acid (Vc), dibutyltin laurate, polyethylene glycol 200 (PEG), hydroquinone, benzoyl peroxide, hydrogen peroxide, ammonium persulfate, and deionized water were among the pure synthetic materials identified via chemical analysis. Ingredients included sodium gluconate, tartaric acid, citric acid, sodium hexametaphosphate, and white sugar, which were all of industrial grade.

### 2.2. Side Chain Synthesis

**Figure 2:**
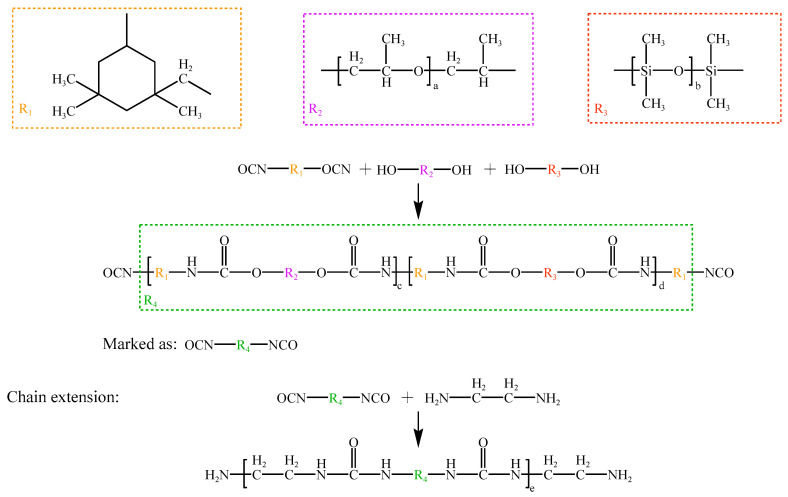
A description of side chain reactions and their molecular structures.

Figure 2 illustrates the molecular structure and synthesis reaction equation of the designed side chain. To enhance the number of small branch chains in the PCE molecules, the backbone was added with polyfunctional compounds which contained amine or hydroxyl groups. As a result, the long branching chains of polyether as well as the short branch chains of chain extenders in alcohol and amine compounds were distributed alternately along its length, enhancing the adaptability and dispersion of the water-reducing agents. The precise process included the following steps:

First, 22.2 g of isophorone diisocyanate was placed in a three-mouth flask, and then added, drop by drop, into the polyethylene glycol 1000 containing dibutyltin dilaurate using a dropping funnel (the solution of dibutyltin dilaurate and dimethyl propionic acid/N-methyl pyrrolidone was 0.2 g per 25 g polyethylene glycol 1000 and 3.35 g dimethyl propionic acid dissolved in 5 mL N-methyl pyrrolidone). A 50 g quantity of water was added next. The mixture of 1,4-butanediol and terminal hydroxy siloxane was mixed in, 2.5 g of ethylenediamine was added, and then 1.5 g of sodium dodecylbenzene sulfonate was added one at a time until the temperature reached 70 degrees Celsius. After that the solution was stirred, while 16% of the free –NCO weight was incorporated into the mixed solution. The solution was removed from heat, and the polyurethane prepolymer was obtained.

### 2.3. Polycarboxylate Preparation

**Figure 3:**
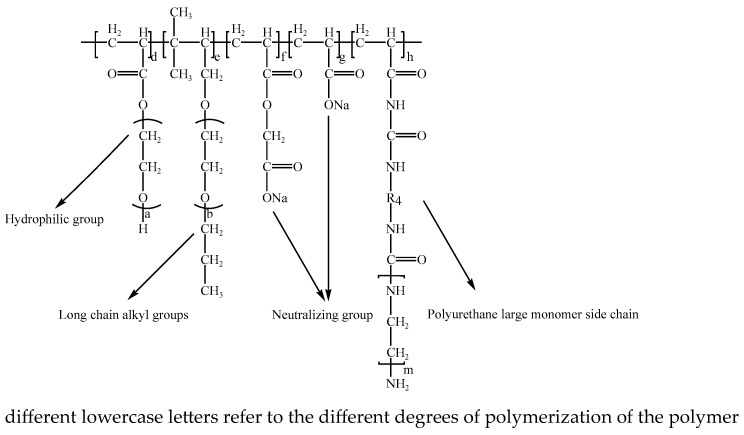
A description of synthesized modified PCE structures.

Figure 3 depicts the intended polycarboxylate molecular structure. In order to ensure an equal molar ratio between acrylic acid and TPEG, as well as to control free radical concentration and maintain the molecular weight regulator, the molar ratio has been changed. In a 250 mL four-mouth flask, 120 g TPEG, 80 mL filtered water, and 10 g PEG were combined and agitated well (until the solution appeared consistent and non-layered). Afterwards, dibutyltin diacetate (0.1 g) and p-methylbenzene sulfonic acid (1.2 g) were added, followed by a small amount of 30% hydrogen peroxide. This solution was used to prepare two components, A and B, as follows:

Component A: (a certain amount of sodium hydrogensulfite and acrylic acid, Vc, or rongalit)

Component B: (a certain amount of Mercaptoacetic acid and ammonium persulfate)

Components A and B were dropped into the beaker using a peristaltic pump at a speed of 2 mL/min, and the process lasted about 2.5 h. After heat preservation for about 1 or 2 h (this range obtained after multiple experiments), the modified polycarboxylate mother solution M was obtained by adjusting the pH by using NaOH to 6–7.

### 2.4. PCE Molecular Weight Test

To create a 1.0 g/L solution, the PCE sample was diluted. The weight-average molecular weight (Mw) and conversion rate of PCE macromonomers were determined using a differential refractive index detector (RID) and gel permeation chromatography (GPC). The mobile phase used for GPC consisted of pure water containing 0.05% sodium azide, with a flow rate of 0.8 mL/min and column temperature maintained at 40 °C.

### 2.5. Determination of PCE Adsorption Capacity

PCE was vibrated in the constant temperature shaker at a set temperature to reach adsorption equilibrium, and it was then pumped and filtered. The obtained filtrate was centrifuged to obtain the supernatant, which was centrifuged twice. The residual concentration of polycarboxylate was measured using a total organic carbon tester (TOC). Then the adsorption capacity of polycarboxylate on the surface of cement particles was calculated based on the following equation.

(1) $$Q_{e}=\frac{(C_{0}-C_{e})V}{M}$$

In this equation, *Q_e_* represents the amount of adsorption (mg/g). *C*_0_ stands for the concentration before the adsorption of the PCE solution (mg/g). *C_e_* means the concentration after the adsorption of the PCE solution (mg/g). *V* is the volume of the solution (the unit was the number 1); additionally, *M* stands for the mass of the cement (g).

The liquor of the water reducer was initially produced in accordance with the planned w/c ratio and PCE percentage. Next, the solution was mixed with gelled materials and stirred via magnetic force for 10 min. The cementitious pastes were separated with a centrifuge for 10 min after being oscillated on the oscillator. The supernatant that had been separated was taken out and put through a 0.45 m nylon microporous filter membrane. In a volumetric flask, the filtrate was transferred and diluted 100 times with deionized water. Liquid TOC from the German company Elementar was used to measure the carbon content of the diluted solution. The concentration of PCE in the solution was determined based on its carbon content, and its adsorption capacity was then determined. The cement content, water-to-cement ratio, and polycarboxylate content were set at 50 g, 0.4, and 0.2%, respectively. 

### 2.6. Surface Tension Measurement

The surface tension of the PCE solution at different concentrations was tested by using A-60 automatic surface tensiometer produced by American Cono Industries Co., Ltd, Boston, MA, USA.

The test of surface tension of cement pastes between the liquid and the solid phases test: A beaker containing 50 g of Portland cement and a set w/c ratio of 0.4 was used. The polycarboxylate ether (PCE) was mixed with 20 g of water and added into the beaker at varying concentrations (0%, 0.1%, 0.2%, 0.3%, 0.4%, and 0.5% by total mass of the materials). The liquid was first mixed slowly for 2 min, followed by vigorous mixing for an additional minute. The resulting cement mixture was then centrifuged and the surface tension of the supernatant was measured.

### 2.7. Flowability

On the basis of GB/T8077-2012 [15], the hollow column model test model was used to test the initial flowability and the net pastes flowability at 1 h and 2 h. The materials included 300 g of Portland cement and a PCE solid content of 0.15%, while the w/c ratio was 0.4. The flowability of the resulting polycarboxylate was measured.

### 2.8. Zeta Potential Value

Polycarboxylate was added to a beaker containing 50 g of gelled material with a water-to-cement ratio of 0.4. The solid concentrations of the PCE mixture were 0, 0.1%, 0.2%, 0.3%, 0.4%, and 0.5% of the mass of the gelled material, respectively. While varying the w/c ratios and polycarboxylate concentrations, we swirled the cement pastes at low speed for two minutes, followed by high speed for one minute. The average of the 5–7 zeta potential measurements performed on each sample over the course of 10 min was recorded.

### 2.9. Self-Shrinkage

The mortar self-shrinkage of the self-compaction cement mortar was measured by using a corrugated pipe and a non-contact probe. The corrugated pipe had an inner diameter of 20 mm and length of 340 ± 5 mm, and the volume transformation of the fluid mortar could be converted into the length transformation. The water-reducing agent concentration in the cement mortar was 0.3–0.5%, and the w/c ratio was 0.3. Free shrinkage experiment, standard sand, benchmark cement, and a 2:1 sand-binder ratio used throughout. Additionally, above 140 mm, cement paste flowability was maintained. For 72 h after casting, the self-shrinkage variations of cement mortar were measured continuously.

### 2.10. Heat of Hydration

TAM Air, an 8-channel standard isothermal calorimeter made in Stockholm, Sweden, was used to quantitatively analyze the total heat release and the heat release rate of the cement pastes with SCMs. A 4 g quantity of gelled material was put into a beaker. A solid content of 0.2% of polycarboxylate was incorporated into the mixture at a water-to-cement ratio of 0.4. The hydration heat of the newly mixed cement samples was tested for 72 h. The temperature in the surrounding area was kept at 20 °C.

## 3. Results and Analysis

### 3.1. The Effects of Initiation Methods on Polycarboxylate Characteristics

**Figure 4:**
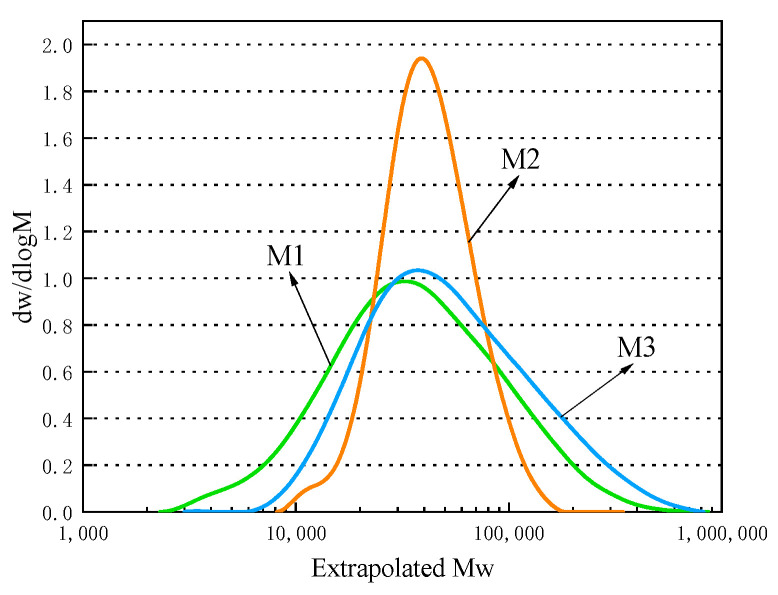
Distribution of the M1, M2, and M3 molecular weights.

Compared to other reaction systems, the REDOX system offers several advantages, including lower activation energy and a faster polymerization rate. The lower activation energy required to initiate polymerization means that the reaction can occur at lower temperatures. Among the three types of composite REDOX systems screened, ammonium persulfate at a mass of 1% of the monomer mass was found to be optimal under typical reaction conditions. Three types of multiple REDOX systems were selected and employed at typical reaction temperatures while a constant mass of ammonium persulfate at 1% of the monomer mass, namely hydrogen peroxide + ammonium persulfate-sodium bisulfite, hydrogen peroxide + ammonium persulfate-Vc, and hydrogen peroxide + ammonium persulfate-rongalit, was maintained. To investigate the properties of polycarboxylate in the three different initiation systems, three types of polycarboxylate named M1, M2, and M3 were synthesized while the other factors were kept constant. Table 2 and Table 3 and Figure 4 present the results of this investigation.

The presented findings provide evidence that, even when a constant TPEG-to-acrylic acid molar ratio is maintained, variation in the initiator system can significantly impact the flowability of cement pastes. The initial flowability values exhibited significant change, and over time, the difference in flowability values between the pastes increased steadily. In particular, the type M1 polycarboxylate demonstrated the lowest surface tension in the hydrogen peroxide + ammonium persulfate-sodium bisulfite initiation system. Regarding both conversion data and molecular weight distribution, type M2 exhibited the most favorable behavior, as the monomer conversion of the molecule was the highest and the molecular weight distribution was the most concentrated. Via analysis, it was determined that the polycarboxylate synthesized at room temperature using the hydrogen peroxide + ammonium persulfate-VC composite initiation system exhibited the best properties.

### 3.2. Orthogonal Experiment

In order to study polycarboxylate synthesis at room temperature, an orthogonal experiment on four components and three levels L_9_(3^4^) was conducted. This experiment examined the law of polycarboxylate produced at ambient temperature and referred to the pertinent literature and experimental experience. The orthogonal table was used to set up the experiment. Numerous experimental settings could be used to identify representative conditions, and fewer trials could be used to infer superior manufacturing conditions. Meanwhile, further statistical analysis could be carried out to obtain more accurate results. Via a single-factor experiment, four primary variables affecting the dispersion characteristics of ethers and polycarboxylates were determined: n(H_2_O_2_), n(TGA), n(H_2_O_2_):n(APS):n(V_C_), n(AA):n(TPEG). A three-level, four-factor orthogonal experiment was designed for this study. Table 4 displays the findings and analyses of the orthogonal experiment. The effects of polycarboxylate, which was produced from the aforementioned components, on the adsorption of Portland cement were examined. The outcomes are displayed in Table 5. 

In order to thoroughly assess how many parameters affect surface tension and saturation adsorption capacity *Q_em_*, the two indices were comprehensively treated to obtain the comprehensive indices, as the table illustrates. The specific methods were as follows: First, the maximum and minimum value of the saturated adsorption capacity *Q_em_* were 100 and 0, respectively. The maximum and minimum value of the surface tension were also 100 and 0. In this way, a centesimal approach might be additionally used to measured values of the two indices. Since the saturated adsorption amount and the weight of surface tension were equal, a comprehensive index was obtained via a 50% weighted average calculation. The average value of the comprehensive indices of all levels referred to K1–K3, and the range means R. Based on the aforementioned results, the optimum synthesis process was A1, D2, C2, B3, which was the same as n(H_2_O_2_) = 0.05 mol, n(TGA) = 0.01 mol, n(AA):n(TPEG) = 2:1, n(H_2_O_2_):n(APS):n(V_C_) = 15:1:1.

The ideal process conditions described above were used to create the polycarboxylate. The reaction took place at a temperature of 40 °C over 4 h. We tested the dispersivity of the polycarboxylate and named it M4; the results are shown in Table 6. Sodium gluconate, citric acid, tartaric acid, sodium hexametaphosphate, and sugar were mixed in a proportion of 3:1:1:1:4, and then the mixture was added into the mother solution for further use, creating a 5% polycarboxylate mother solution.

### 3.3. Effects on the Surface Tension of the Admixture Content

**Figure 5:**
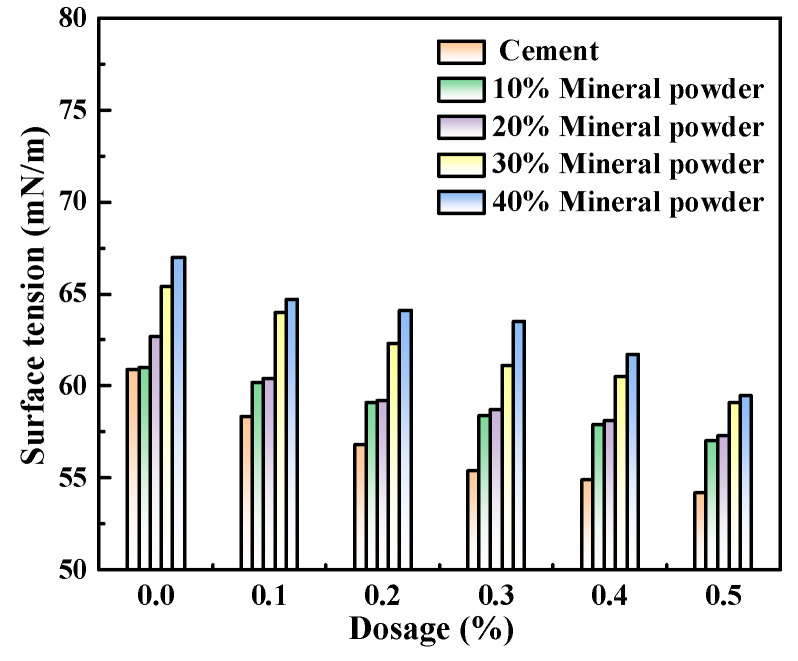
M4 and mineral powder dosage effects on the liquid phase surface tension.

**Figure 6:**
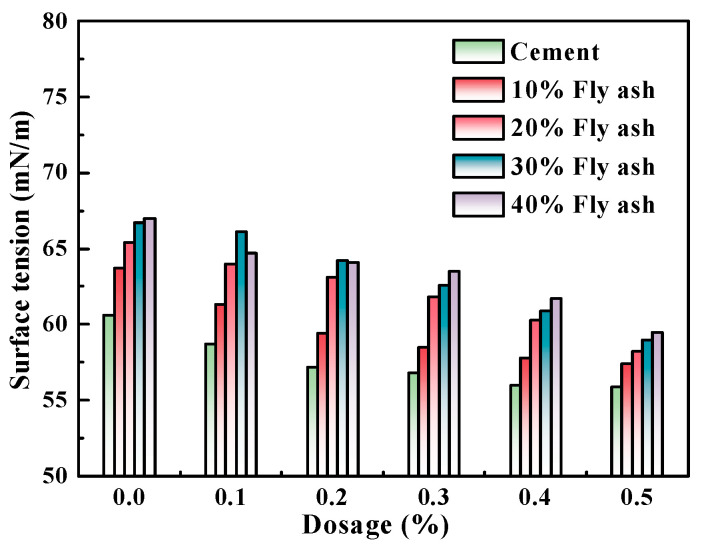
M4 and fly ash dosage effects on the liquid phase surface tension of cement pastes.

**Figure 7:**
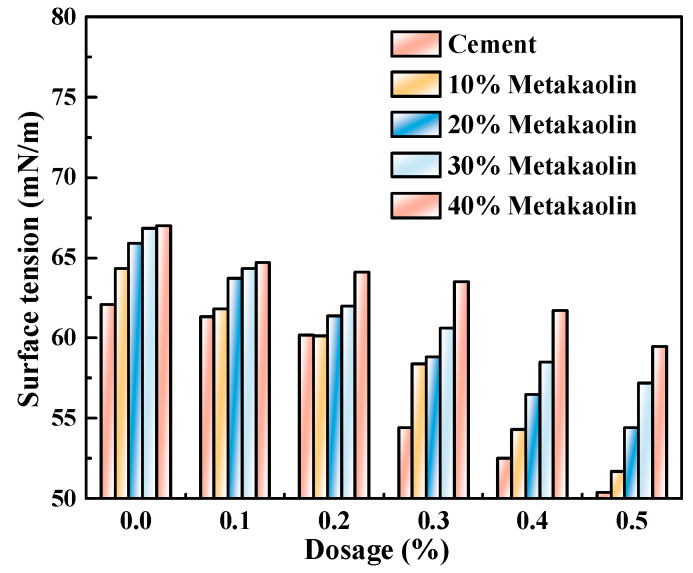
M4 and metakaolin dosage effects on the liquid phase surface tension of cement pastes.

According to the results in Section 3.2, M4, with the best performance, was selected to prepare the cement pastes with a w/c ratio of 0.4. In the mixture, the masses of mineral powder, fly ash, and metakaolin that substituted for Portland cement were 0, 10%, 20%, 30%, and 40%, respectively, and the admixture contents of the water-reducing agent were 0, 0.1%, 0.2%, 0.3%, 0.4%, and 0.5%. The newly mixed cement paste was first obtained via centrifugal separation, and then its liquid-phase surface tension was tested. Figure 5, Figure 6 and Figure 7 show the influence of different admixture content of M4 and SCMs in relation to the surface tension of fresh cement pastes.

Similar to below, the w/c ratio was 0.4.

Figure 5, Figure 6 and Figure 7 show that as the admixture content of M4 increased, the liquid-phase surface tension of newly mixed cement pastes progressively decreased, with the minimum reduction reaching 11.19%. This indicates that with the increase in the admixture content of M4, the liquid-phase surface tension was observed to decrease as the number of unabsorbed polycarboxylate molecules in the liquid phase increased. 

The addition of various cementitious ingredients led to a decrease in the surface tension of the produced pastes. The surface tension further decreased with increasing admixture content. This can be attributed to the smaller particle size of the mixed mineral powder and fly ash compared to Portland cement. These materials consist of aluminosilicate glass beads and sponges that resemble glass beads, with a small average particle size and internal specific surface area. Aluminosilicate glass beads exhibit advantages such as dense texture, smooth surface, good flowability, and reduced adsorption of free water, thus acting as “ball bearings” in the concrete mixture. These products effectively fill the voids left by Portland cement hydration products and can significantly reduce internal porosity, resulting in refinement of the pore size.

After the addition of the SCMs, the concentrations of plasma Ca^2+^, Na^+^, and OH^−^ in the liquid phase decreased due to the lower solubility of the admixture compared to Portland cement particles. During the early hydration of Portland cement, the Si-O bond would break in the alkaline environment, and then combine with hydrated and dissociated ions to form C–S–H gels. This caused a decrease in the concentration of reverse ions on the surface of Portland cement particles, which in turn influenced the liquid-phase surface tension [16,17,18,19]. 

### 3.4. Effects on the Flowability of the Admixture Content

**Figure 8:**
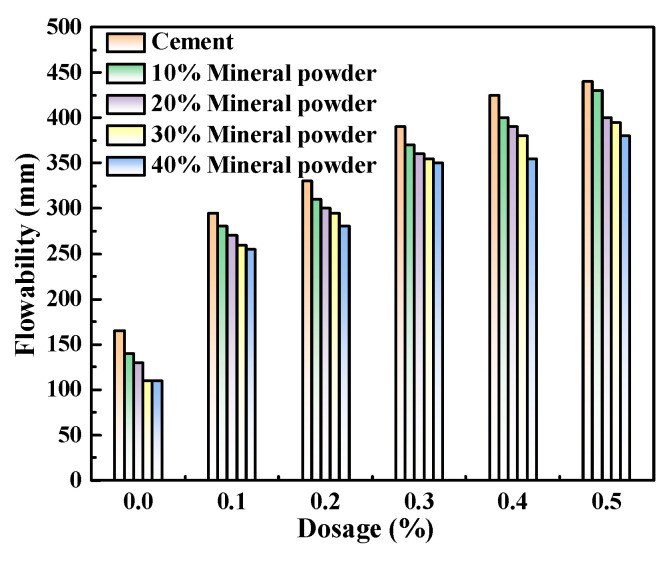
M4 and mineral powder dosage effects on cement pastes flowability.

**Figure 9:**
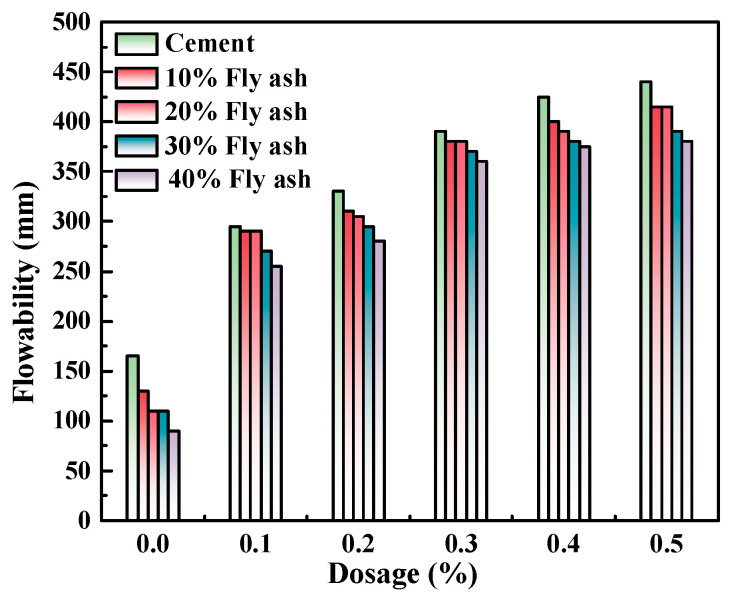
M4 and fly ash dosage effects on cement paste flowability.

**Figure 10:**
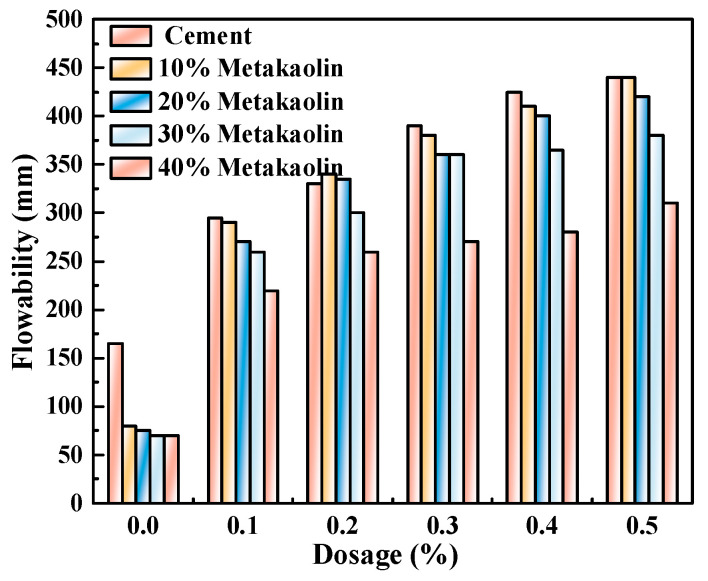
M4 and metakaolin dosage effects on cement paste flowability.

Figure 8, Figure 9 and Figure 10 illustrate the flowability of fresh cement pastes at a w/c ratio of 0.4 with varying admixture contents of polycarboxylate and SCMs. As depicted, the initial flowability of pure Portland cement pastes and those mixed with three SCMs consistently increased with the increasing content of M4 admixture, reaching a maximum increase of 342.8%. The rise in flowability may account for the enhanced adsorption capacity of Portland cement and various SCMs as the content of polycarboxylate superplasticizer admixture increased, thereby enhancing the free water capability and flowability of the system. Moreover, the addition of mineral powder, fly ash, and metakaolin under identical experimental conditions led to a decrease in the initial flowability of the fresh cement pastes to varying degrees, with metakaolin exerting the most significant influence, followed by mineral powder and fly ash.

In previous studies, the impact of incorporating various SCMs on rheological properties adsorption that of cement has been tested, and the results indicate that an increase in the SCMs content led to a continuous decrease in the rheological properties of the cement [20,21,22,23]. However, the mechanisms of these three materials exhibit slight differences.

Mineral powder, being mainly composed of vitreous material, had an irregular shape similar to that of Portland cement particles. Its crystallinity was low, and its particle arrangement was disordered, resulting in a relatively large specific surface area. After adding the admixture, the specific surface area of the cement pastes containing mineral powder increased, leading to more adsorption of polycarboxylate superplasticizers by the mineral powder in the pastes [24,25]. Consequently, the number of polycarboxylate molecules adsorbed on Portland cement particles decreased, making it difficult for the particles to slide against each other, resulting in a reduction in the flowability of the cement pastes.

Fly ash is characterized by its spherical shape, loose structure, and microporosity, which make it highly adsorbable. Fly ash added to cement paste effectively adsorbs polycarboxylate molecules, and the spherical particles of fly ash can effectively fill in the gaps between Portland cement particles, leading to a more compact structure and smaller interparticle spacing [26]. Consequently, both the friction and adhesion interactions between the particles are strengthened, making it more difficult for the Portland cement particles to slide against each other. This results in increased resistance to deformation, and the flowability of the cement paste is observed to decrease significantly after the addition of fly ash.

After being calcined at high temperatures, the original 1:1 shape of the metakaolin dioctahedral silicate structure is transformed into a debris topography consisting of lamellar octahedral alumina and other components, resulting in a transition phase with relatively poor crystallinity. This process leads to the disappearance of the accumulation phenomenon and an increase in interparticle pore size. The distinctive interlayer structure of metakaolin facilitates the adsorption of water and polycarboxylate molecules [27,28], resulting in a decrease in the free water content of metakaolin-mixed cement pastes along with a reduction in the quantity of polycarboxylate molecules required to disperse Portland cement particles. Consequently, the flowability of the pastes decreases significantly with an increase in metakaolin admixture content.

### 3.5. Effects on the Zeta Potential of the Admixture Content

**Figure 11:**
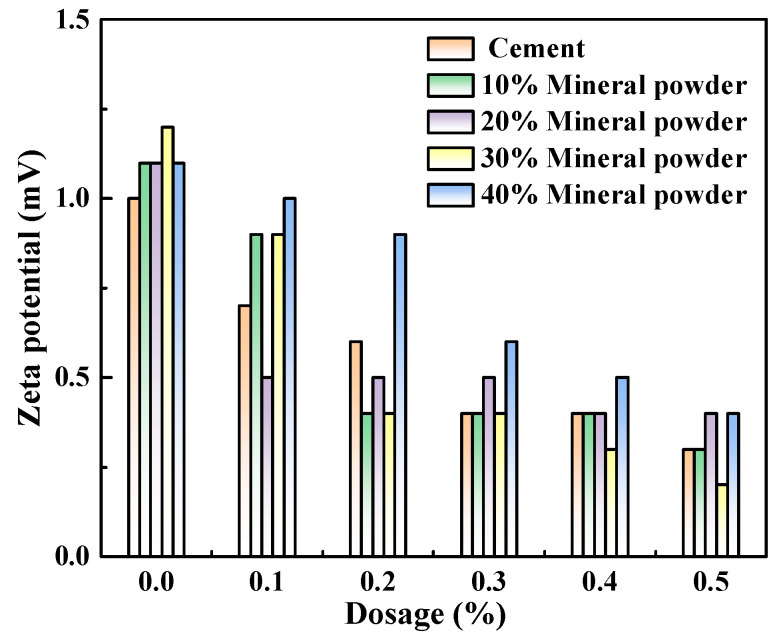
M4 and mineral powder dosage effects on zeta potential of cement pastes.

**Figure 12:**
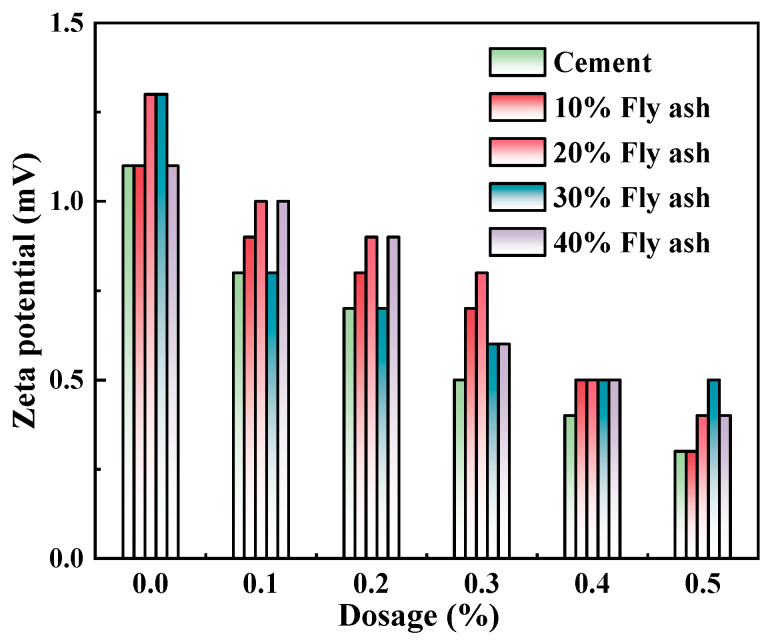
M4 and fly ash dosage effects on zeta potential of cement pastes.

**Figure 13:**
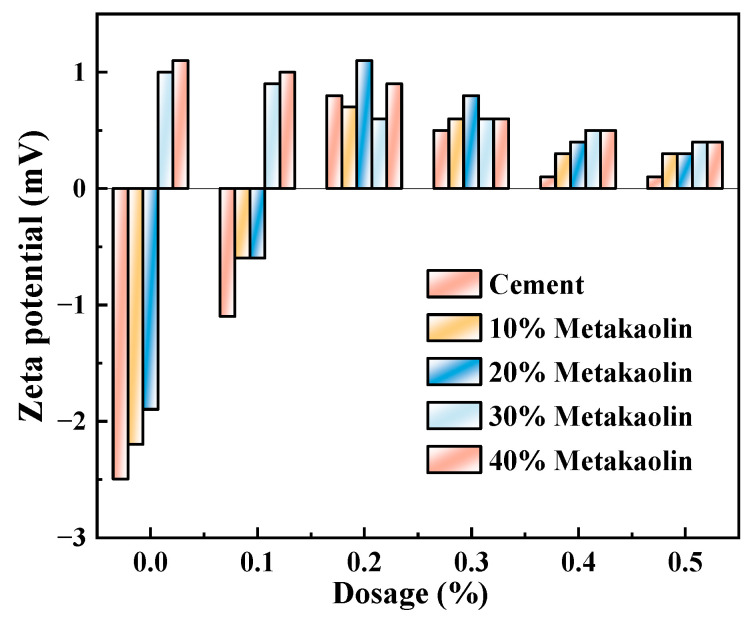
M4 and metakaolin dosage effects on zeta potential of cement pastes.

The zeta potential of cement pastes mixed with M4 was investigated with varying levels of SCM content (0%, 10%, 20%, 30%, and 40%) and water-reducing agent content (0%, 0.1%, 0.2%, 0.3%, 0.4%, and 0.5%) while maintaining a constant w/c ratio of 0.4, as presented in Figure 11, Figure 12 and Figure 13. As demonstrated in Figure 8, Figure 9 and Figure 10, the addition of metakaolin, mineral powder, and fly ash all have a negative impact on the dispersion of Portland cement, with the extent of the impact decreasing in the order of metakaolin, mineral powder, and fly ash. The influence on the dispersion of Portland cement with fly ash or mineral powder is similar.

The surface potential of the particles underwent changes during the process of Portland cement hydration, with corresponding changes to the potential of the diffusion layer. The maintenance of electrostatic repulsion was crucial to maintaining zeta potential. The adsorption of polycarboxylate superplasticizers onto cement particle surfaces led to the extension of functional group-polyoxymethylene chain segments into the solution. This resulted in the formation of hydrogen bonds with H_2_O molecules in both pure Portland cement pastes and those blended with the three supplementary cementitious materials. The formation of a solvent hydration shell resulted in lubrication and steric hindrance effects [29]. These effects were the main functions of the polycarboxylate superplasticizers.

In a gel system, zeta potential reflects the potential of double electrode layers; additionally, between molecules, it has a significant correlation with electrostatic repulsion. From the Debye–Huckel formula:

(2) $$\frac{1}{F_{\mathrm{ES}}}=\frac{-1}{2\pi \varepsilon \varepsilon_{0}\bar{a}\psi^{2}}\times K^{-1}\times [e^{k(h-2l)}+1]$$

In this equation, *F*_ES_ stands for electrostatic repulsion, *ε* represents the relative dielectric constant of water, *ε*_0_ is the vacuum dielectric constant, $\bar{a}$ refers to the mean radius of particles, *ψ* represents electrodynamic potential, *K* stands for the Debye–Huckel parameter, *h* denotes the distance between two particles, and *l* indicates the thickness of the adsorption layer. PCE follows Langmuir’s monolayer adsorption law on the outside of particles of Portland cement [30]. It can be seen from Equation (1) that with the increase to the admixture content of the water-reducing agent, the thickness of adsorption layer *l* remains unchanged, and the distance between two particles *h* is decreased. In this way, the electrostatic repulsion force *F*_ES_ is decreased [31]. The stability of the system is decreased, so zeta potential value drops. In addition, a large number of anionic groups are contained in the mixed polycarboxylate superplasticizer, influencing the charge distribution of Stern double electrode layers on the localized surface of Portland cement particles. In the experiment, a large difference manifested between the ion concentration on the Portland cement particle surface and the main ion concentration in the pastes. The counter ion accumulation near the surface layer shielded the surface charge, thus reducing zeta potential. Therefore, with the increase of the admixture content of M4, the absolute value of zeta potential showed a decreasing trend. Similar results were also obtained in the literature [32]. 

It is evident that the zeta potential in cement pastes decreased when the admixture content of polycarboxylate superplasticizer was 0–0.2% and the three SCMs, namely fly ash, mineral powder, and metakaolin, were added. This decrease in zeta potential is one of the main reasons for the reduction in flowability and liquid-phase surface tension of the newly mixed cement pastes. The solubility of Portland cement particles is higher than that of mineral powder and fly ash, resulting in an increase in K^+^, Ca^2+^, and Na^+^ plasma concentrations in its liquid phase [17]. Mineral powder, which is made up of irregular particles similar in shape to Portland cement particles, has a very low degree of crystallization and exists mostly in the form of glass phases. Fly ash, on the other hand, is composed of spherical particles with a fluffy structure, many broken bonds, and micropores, which allows it to effectively adsorb H_2_O molecules in the pastes. The addition of these two SCMs reduces the amount of Portland cement in the mixture, leading to a decrease in the total production of hydration products, especially Ca (OH)_2_ and hydrated calcium silicate, which play a crucial role in improving zeta potential. As a result, the stability of the pastes decreases, and zeta potential also decreases correspondingly [33]. 

When metakaolin is added, the hydroxy (OH^−^) is removed at the calcination temperature of 600–800 °C, causing the initial 1:1 shape of dioctahedral silicate to break down and degrade into a lamellar octahedral fragment morphology with low crystallinity, producing an Al_2_O_3_–SiO_2_ transition phase. This structural change causes amorphous Al_2_O_3_–SiO_2_ to have very high pozzolanic activity, and metakaolin has particularly strong adsorption of H_2_O [34]. Consequently, the addition of metakaolin significantly reduces the quantity of hydration products produced by Portland cement, leading to a decrease in the absolute value of zeta potential.

Additionally, in the absence of polycarboxylate superplasticizer addition, it was observed that increasing the admixture content of metakaolin from 20% to 30% resulted in an increase in zeta potential from −1.9 mV to 1.0 mV. Furthermore, as the admixture content of polycarboxylate superplasticizer increased, the negative zeta potential value of the combination of metakaolin and cement pastes became positive. This was due to the Al–OH bond breaking and becoming positively charged on the faceted pebble of the metakaolin lattice edge. However, factors such as lattice defects and isomorphic substitution (where Ca^2+^ and Mg^2+^ replaced Al^3+^ on the lattice) resulted in a negative charge at the crystal level of the metakaolin. After coming into contact with water, metakaolin formed a constant charge surface, which was positively charged at the particle edge and negatively charged at the crystal level. The zeta potential values of metakaolin were determined from the presence of ions with opposite charges on the shear plane during solid-liquid sliding. Under alkaline conditions, these ions were negatively charged [35]. As a result, metakaolin had little effect on Portland cement at an admixture content of 10%. However, with an increase in admixture content, the zeta potential of cement pastes increased from negative to positive. The greater the admixture content, the stronger the electronegativity. Furthermore, with an increase in the admixture content of polycarboxylate superplasticizer, the hydration products of C_3_A and C_4_AF in Portland cement correspondingly increased. As they were positively charged, the zeta potential became positive [36], gradually masking the influence of metakaolin. This explains why the zeta potential changed.

The Al–OH bond broke and became positively charged on the faceted pebble at the metakaolin lattice edge. However, due to factors including lattice defects and isomorphic substitution (Ca^2+^ and Mg^2+^ replaced Al^3+^ on the lattice), metakaolin was negatively charged at the crystal level. After contact with water, metakaolin formed a constant charge surface, which was positively charged at the particle edge and negatively charged at the crystal level. The reverse-sign ions on the shear plane of the solid-liquid slide determined the positive and negative zeta potentials of metakaolin. Under alkaline conditions, the reverse-sign ions were negative and negatively charged [35]. Therefore, when the admixture content of metakaolin was 10%, it had little influence on Portland cement, but with the increase of the admixture content, the zeta potential of cement pastes was elevated to positive from negative. The higher the admixture content, the stronger electronegativity. With the increase in polycarboxylate superplasticizer content in the admixture, the hydration products of C_3_A and C_4_AF in Portland cement increased correspondingly; as they were positively charged, the zeta potential also became positive [36], gradually shielding the influence of metakaolin, which is the reason why the zeta potential changed.

**Figure 14:**
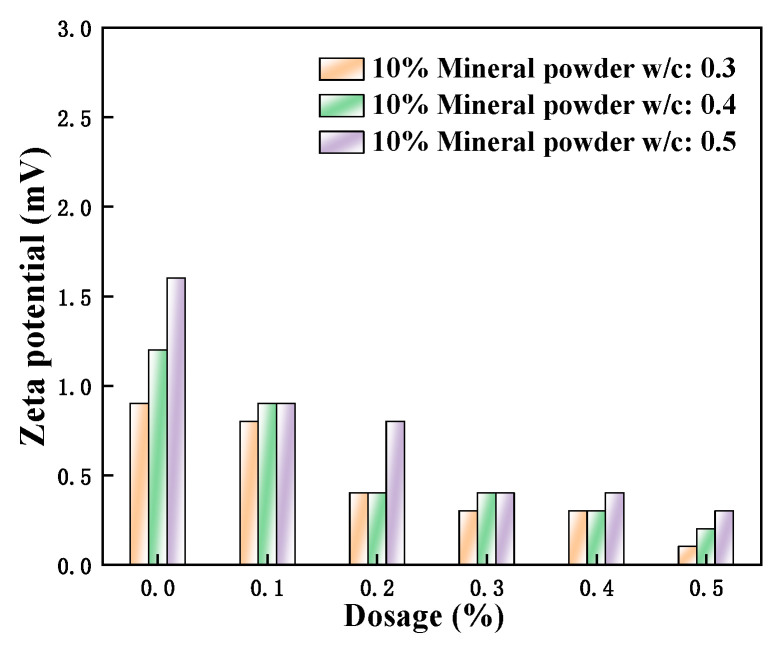
Effect of M4 dosage and w/c ratio on zeta potential of cement pastes with 10% mineral powder.

**Figure 15:**
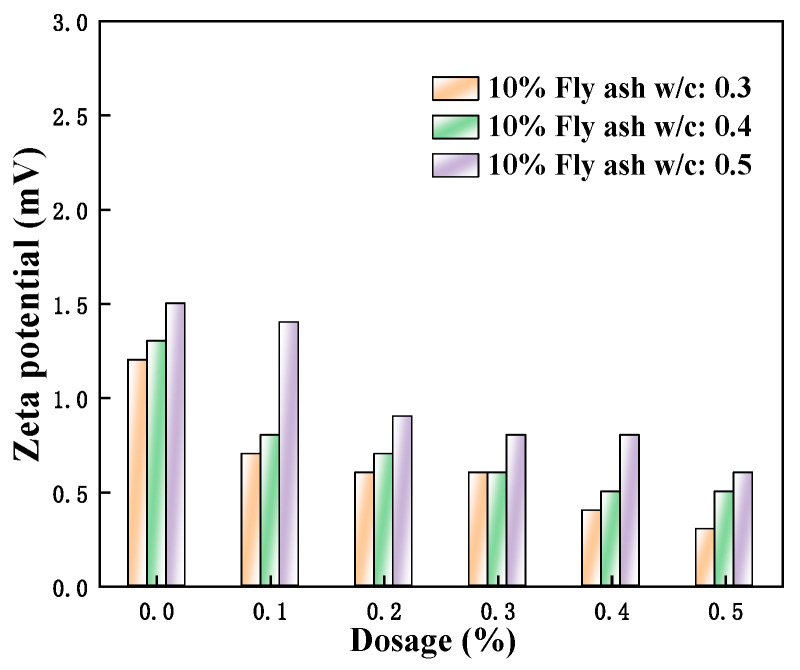
Effect of M4 dosage and w/c ratio on zeta potential of cement pastes with 10% fly ash.

**Figure 16:**
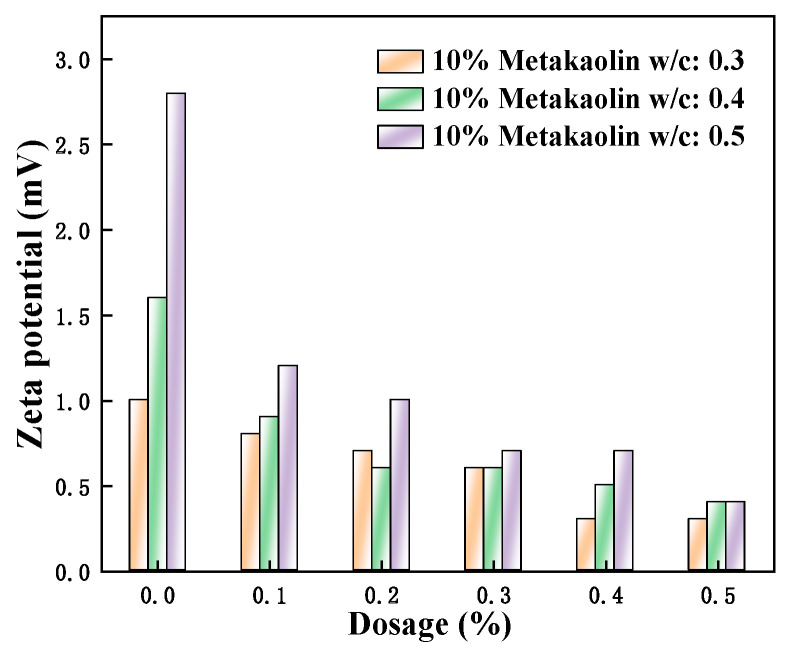
Effect of M4 dosage and w/c ratio on zeta potential of cement pastes with 10% metakaolin.

Figure 14, Figure 15 and Figure 16 show the zeta potential of the cement pastes mixed with fly ash, metakaolin, and mineral powder at a content of 10% when the w/c ratios of 0.3, 0.4, and 0.5 were used; additionally, the amount of PCE is 0, 0.1%, 0.2%, 0.3%, 0.4%, and 0.5%. It can be seen that the zeta potential values of the cement pastes mixed with different SCMs are all positive. The primary minerals that make up Portland cement, cause the particles in hydrated pastes of C_2_S and C_3_S to have a negative charge, whereas those in C_3_A and C_4_AF have a positive charge. The solubility of silicate is not as good as aluminate. Hence, the zeta potential displayed a positive value, as the positively charged hydration products dominated [36]. 

Although a certain amount of anionic polycarboxylate superplasticizer was added, the zeta potential exhibited a decreasing trend on the surface of cement particles. At this time, the w/c ratio was flat, and the admixture content was as well. Therefore, they were insufficient to affect the positive potential of the hydration products obtained from aluminate, and the measured zeta potential was positive. The results also showed that the zeta potential had increased to varying degrees with the decrease of w/c ratios. According to Degenne’s steric hindrance ratio theory:

(3) $$F_{\mathrm{Stern}}=\bar{a}\times \frac{6\pi K_{B}T}{S^{2}}\left[{\left(\frac{2L}{h}\right)}^{5/3}-1\right]$$

In this equation, *F*_Stern_ stands for the acting force of the steric hindrance and represents the mean radius of particles. *K*_B_ stands for the Boltzmann constant. *T* means the absolute temperature. *S* denotes the distance between the centroids of two adjacent mushroom-like polymer clusters. *L* represents the maximum distance of the high polymer extending into the solvent. *h* is the distance between the two particles.

With the decrease of the w/c ratio, either the *S* decreased or the spatial resistance *F*_Stern_ increased. At this time, the stability of the system improved. The minification of the w/c ratio caused the slow dissolution of the cement surface, and neither did the ion concentration increase in the liquid phase. As the thickness of the Stern double electric layer increased, the corresponding zeta potential was also greater. Additionally, when the w/c ratio decreased and the consistency of the pastes increased during the hydration process, the stability of the pastes became better and better. Moreover, the electrolyte concentration became higher, and the zeta potential also increased correspondingly [37]. When the w/c ratio increased, the zeta potential decreased slightly with continuous hydration. This was because the new hydration products were constantly generated during the Portland cement hydration. Some electrolyte ions were absorbed by Portland cement particles with opposite charges, and the directed migration of electrolyte ions was not easy under the influence of the outside weak electrical field. Accordingly, the measured zeta potential values of the cement pastes that were mixed with mineral powder, fly ash, and metakaolin did not decrease significantly. 

### 3.6. Effects on the Self-Shrinkage of the Admixture Content

**Figure 17:**
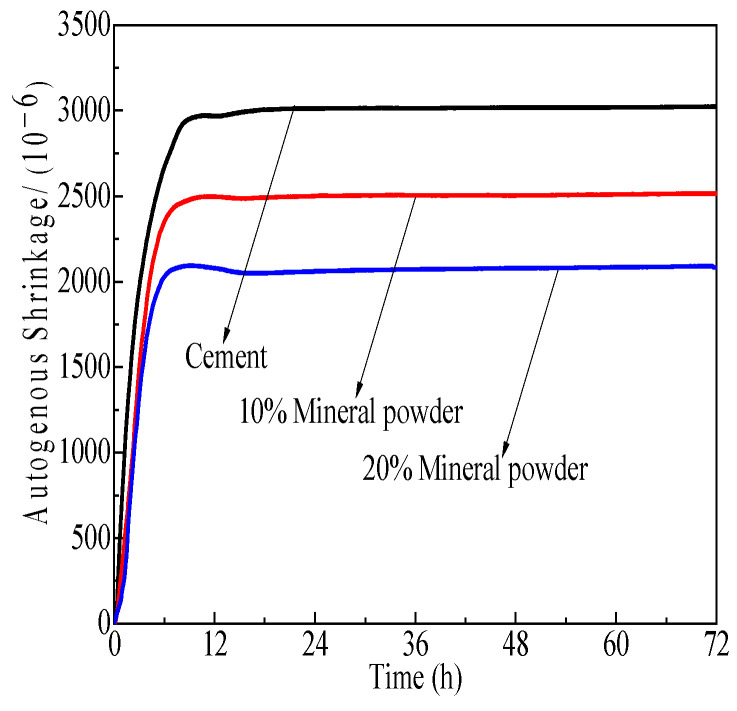
Effects of M4 and mineral powder content effects on cement paste self-shrinkage.

**Figure 18:**
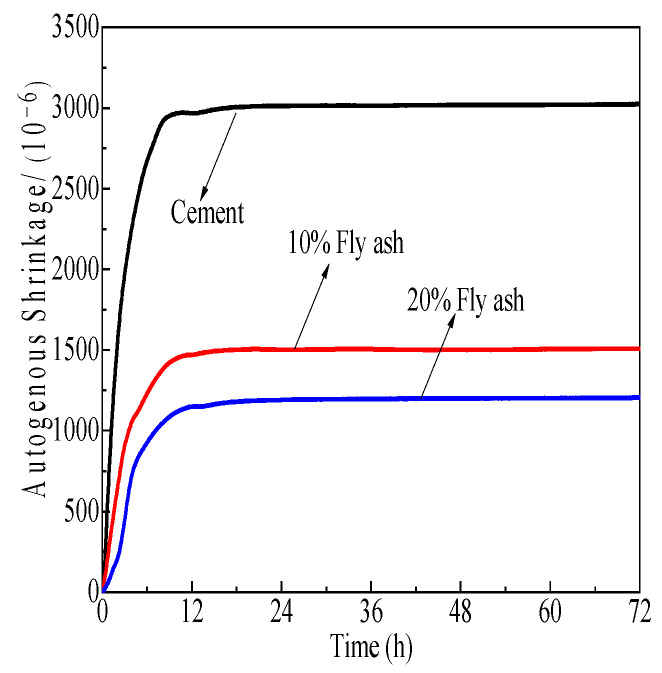
Effects of M4 and fly ash content on cement paste self-shrinkage.

**Figure 19:**
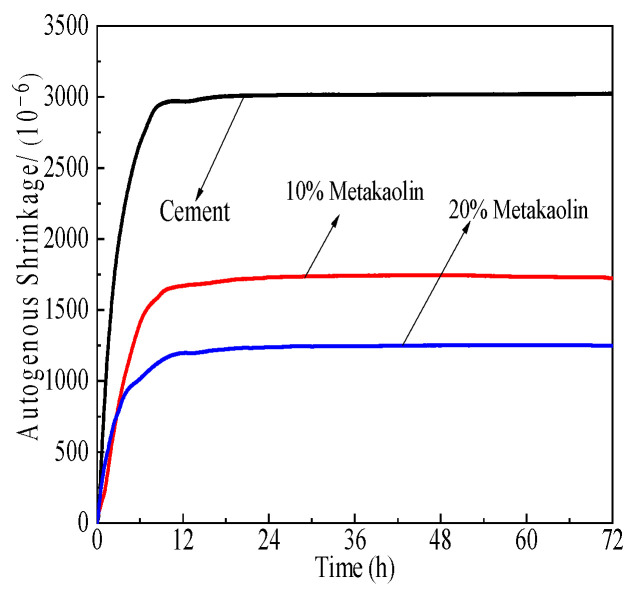
Effects of M4 and metakaolin content on cement paste self-shrinkage.

Figure 17, Figure 18 and Figure 19 demonstrate the effect of self-shrinkage on cement pastes with a water-to-cement ratio of 0.3 and varying concentrations of SCM polycarboxylate admixtures, namely 0%, 10%, and 20%. The results demonstrate that the incorporation of mineral powder, fly ash, and metakaolin to the mixture has a moderating effect on self-shrinkage. Specifically, fly ash exerts the most substantial impact, followed by metakaolin and mineral powder.

Figure 17, Figure 18 and Figure 19 depict the impact of SCMs, including metakaolin, fly ash, and mineral powder, on the degree of shrinkage in cement pastes when the admixture concentration of polycarboxylate is varied at 0%, 10%, and 20% and the w/c ratio is 0.3. The results indicate that self-shrinkage modifications in cement pastes are mostly complete within 12 h of hydration under experimental conditions. The addition of these cementitious materials significantly reduces the self-shrinkage tendency of Portland cement, with the maximum reduction rates achieved at 31.1%, 60.1%, and 57.4% for mineral powder, fly ash, and metakaolin, respectively. Fly ash and metakaolin exhibit a more pronounced effect in reducing self-shrinkage. Mineral powder, with its lower activity, replaces part of the cement and thus weakens the self-drying effect, leading to a decrease in self-shrinkage, which increases with the increase in admixture content. Fly ash reacts slowly in the early stages of cement hydration and makes a limited contribution to the formation of hydration products, which are dominated by Portland cement. Nevertheless, the presence of fly ash particles inhibits chemical shrinkage to some extent. Metakaolin, despite its high reactivity, leads to a significant rise in early hydration products, accompanied by intense heat release. However, cement pastes exhibit reduced self-shrinkage due to thermal swelling from hydration products.

### 3.7. Effects on the Hydration Heat of the Admixture Content

The hydration heat graph of cement pastes is shown in Figure 20, Figure 21, Figure 22, Figure 23, Figure 24, Figure 25, Figure 26 and Figure 27 at 0 and 10% admixture concentrations of extra cementitious materials and 0, 0.1%, 0.3%, and 0.5% admixture levels of polycarboxylate.

**Figure 20:**
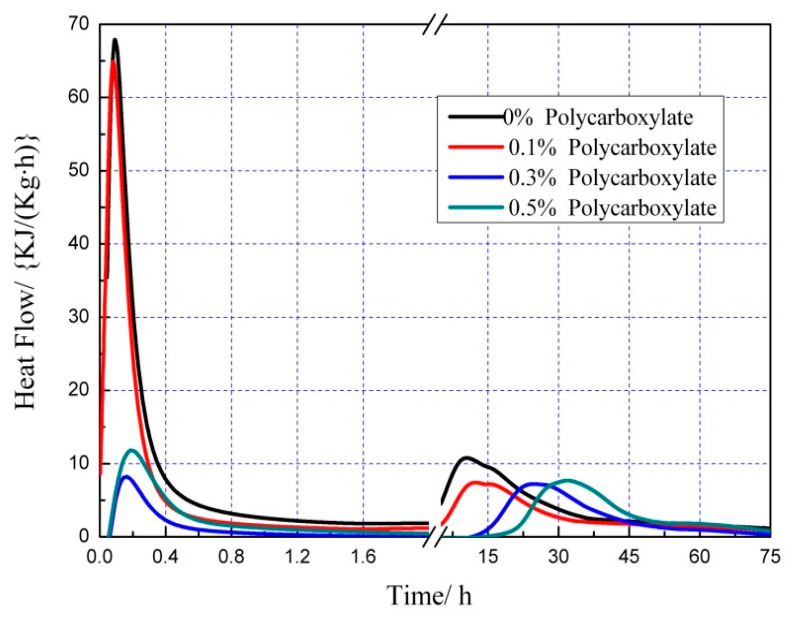
Effect of M4 dose on hydration heat flow of cement pastes.

**Figure 21:**
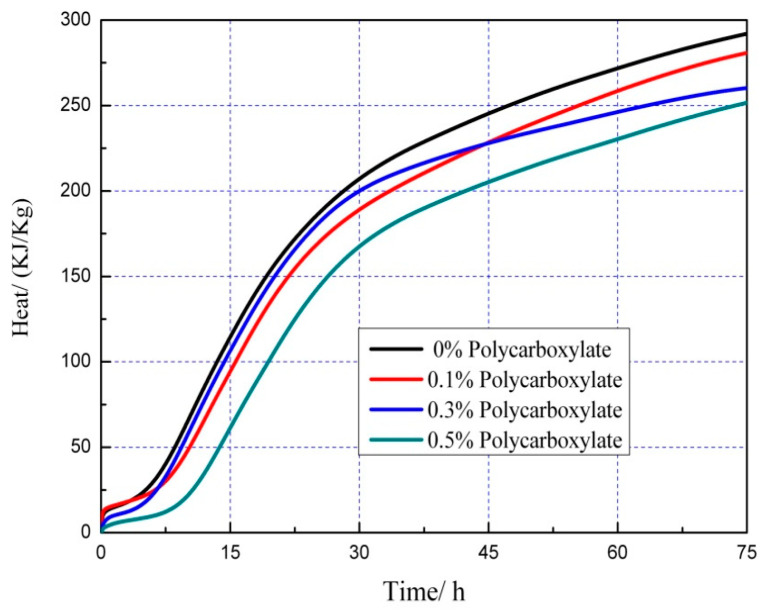
Effect of M4 dose on hydration heat of cement pastes.

**Figure 22:**
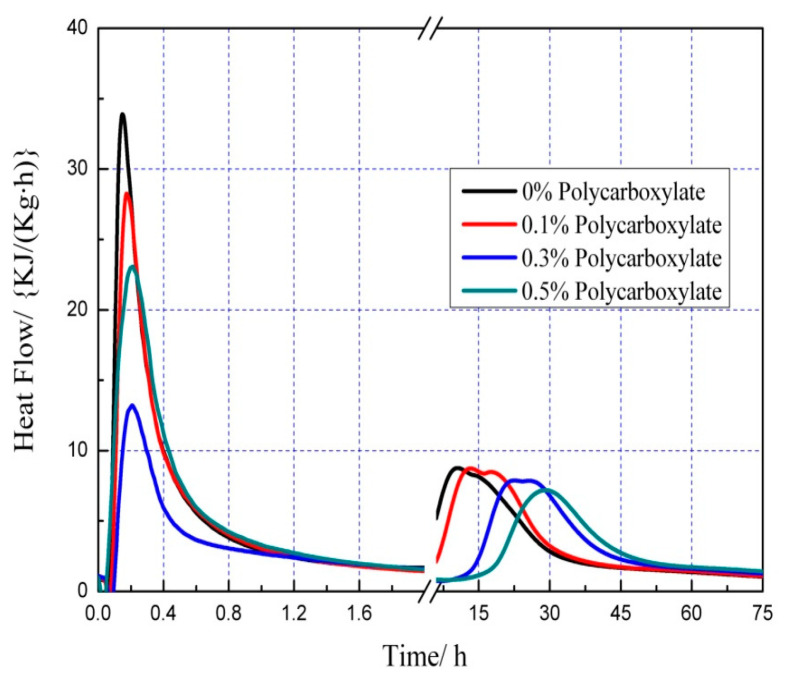
Effect of M4 dosage on hydration heat flow of cement pastes of 10% mineral powder.

**Figure 23:**
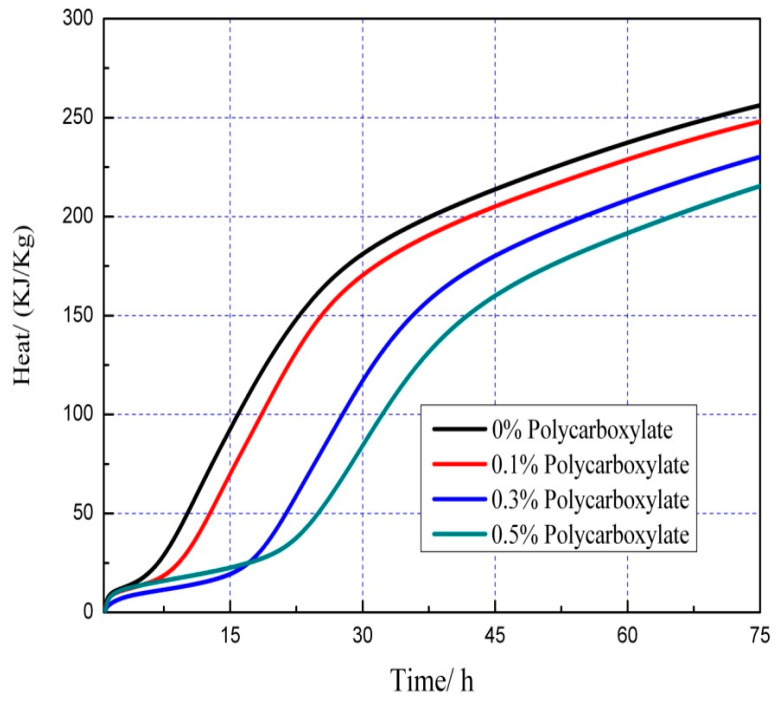
Effect of M4 content on hydration heat of cement pastes of 10% mineral powder.

**Figure 24:**
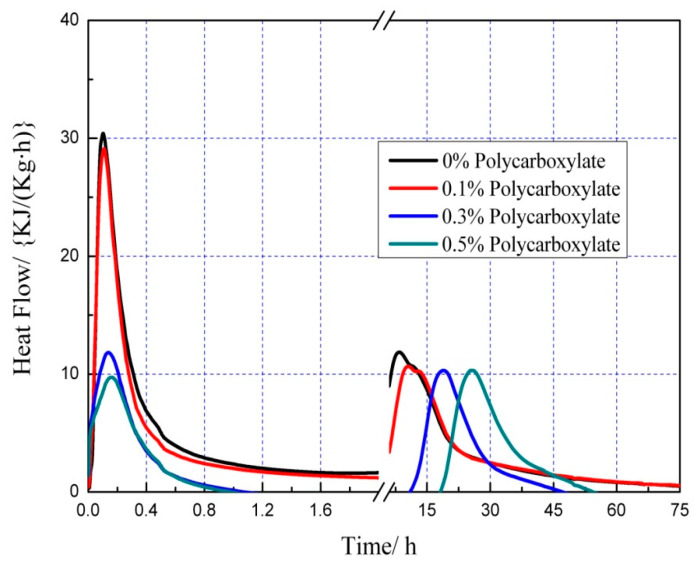
Effect of M4 content on hydration heat flow of cement pastes of 10% fly ash.

**Figure 25:**
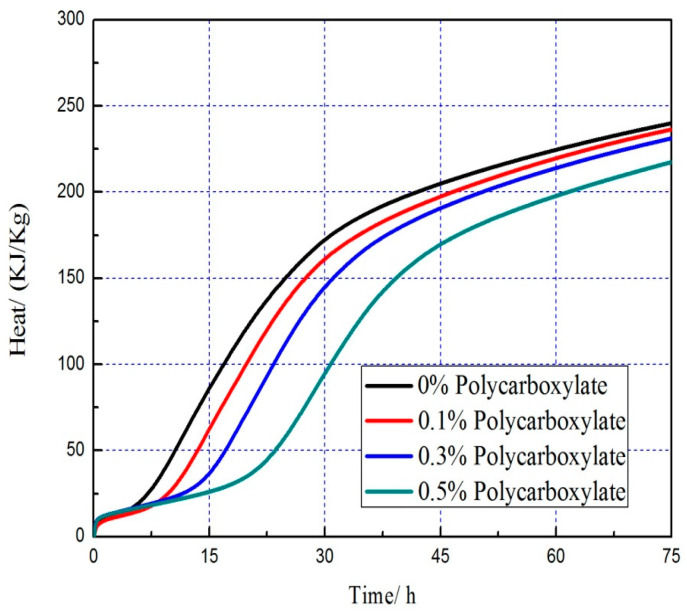
Effect of M4 content on hydration heat of 10% fly ash cement pastes.

**Figure 26:**
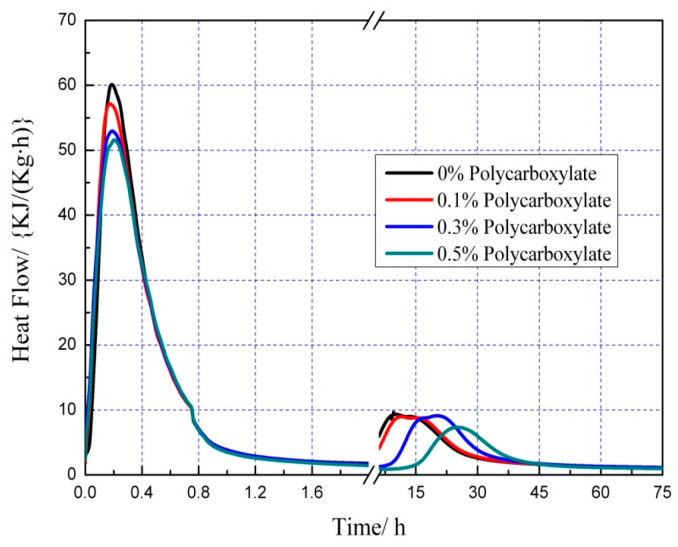
Effect of M4 content on hydration heat flow of 10% metakaolin cement pastes.

**Figure 27:**
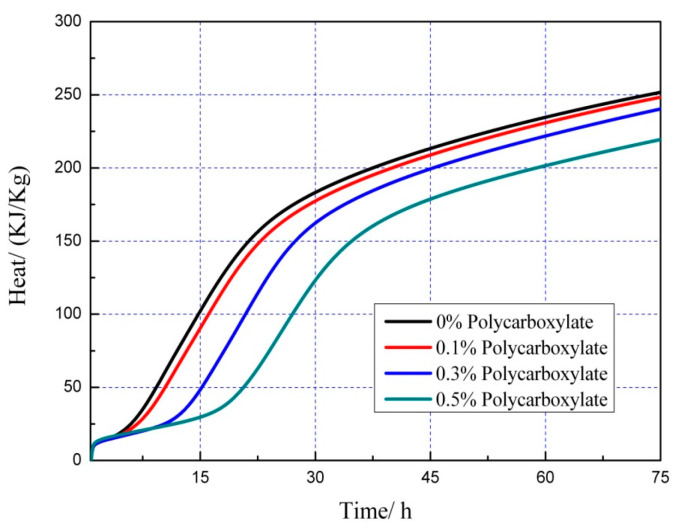
Effect of M4 content on hydration heat of 10% metakaolin cement pastes.

Based on the findings presented in Figure 20, Figure 21, Figure 22, Figure 23, Figure 24, Figure 25, Figure 26 and Figure 27, it was observed that M4 superplasticizer exhibited a superior performance in reducing overall heat release during cement hydration and in slowing down the heat release rate. The effectiveness of this compound became more evident with increasing concentrations. This could be attributed to the presence of sugar in the mixture, which caused surface activity that coated the Portland cement particles with a film layer, delaying the hydration process. Additionally, the adsorption of the superplasticizer caused significant changes to the morphology of cement particle surfaces during the early stages of hydration. The active groups of the superplasticizer combined with hydration-generated ions to form unstable complexes, hindering both the precipitation and the hydration process of the initial phases in an alkaline solution. The hindrance effect increased with an increase in admixture content, causing a decrease of the dissolution peak. The effects of polycarboxylate side chains on Portland cement, such as adsorption, complexation, and delayed coagulation, hindered the hydration process of mineral compounds and slowed down their growth. The speed at which the hydration energy barriers were overcome was also notably reduced, causing a delay in both the hydration growth during the induction phase and the second exothermic peak. As a result, a rightward shift of the second exothermic peak was observed, clearly demonstrating the delay effect.

Furthermore, the incorporation of supplementary cementitious materials can decrease heat release during the hydration process of Portland cement under identical conditions. The rapid dissolution of free lime in SCMs causes the system to release heat quickly and leads to the formation of ettringite (AFt). The hydrated calcium sulfoaluminate coating the cement particles hinders the interaction of water with cement, and the heat release rate begins to increase during the subsequent process. The coating layer is destroyed under pressure, which increases the reaction rate of water and cement, leading to the maximum heat release. By replacing part of the Portland cement, the amount of clinker in the cement is reduced, resulting in a decrease in the overall heat release. During the induction and acceleration periods of the second and third stages of hydration, the total heat release decreases [26]. 

## 4. Conclusions

1. The polycarboxylate molecules produced by the REDOX system of hydrogen peroxide+ammonium persulfate-V_C_ exhibited a compact high monomer conversion rate and molecular weight distribution when compared to other reducing agents such as sodium bisulfite and rongalit. This led to excellent overall performance, including longer side chains and improved steric hindrance and dispersion compared to regular PCE. The dispersibility of the synthesized polycarboxylate was found to be improved with an increase in the molar ratio of AA/TPEG under identical experimental conditions. The optimal synthesis process was determined to be at room temperature using TGA and H_2_O_2_ molar masses of 0.01 and 0.05, respectively, with AA and TPEG in a 2:1 molar ratio and an H_2_O_2_ to APS to Vc ratio of 15:1:1.
2. As the content of SCMs and polycarboxylate superplasticizer admixture increased, there was a decrease in the surface tension of cement pastes when combined with metakaolin, fly ash, and mineral powder. This decrease in surface tension also resulted in a decrease in the stability of the cement pastes, as well as a decrease in the zeta potential value.
3. The zeta potential of cement pastes increased upon addition of supplementary cementitious materials and reduction in w/c ratio, which resulted in improved steric hindrance effect and system stability when mixed with mineral powder, fly ash, and metakaolin. This effect was accompanied by an increase in the paste consistency and a more noticeable change in zeta potential.
4. As a result of its unique lamellar structure and smaller particle size, metakaolin exhibits a stronger adsorption effect on water and polycarboxylate superplasticizer than Portland cement. Therefore, the inclusion of metakaolin had a greater impact on the surface tension, flowability, and zeta potential of cement pastes.
5. In cement pastes mixed with polycarboxylate superplasticizer (w/c = 0.3), the addition of mineral powder, fly ash, and metakaolin slowed down its self-shrinkage, with the most significant effect observed with fly ash, followed by metakaolin and mineral powder. Additionally, increasing the content of PCE resulted in a further reduction in both overall heat release and heat release rate.

## Figures and Tables

**Table 1:** Chemical composition of metakaolin, mineral powder, fly ash, and PI 42.5 Portland cement, w/%.

Material	Chemical Composition w/%										
	SiO_2_	Al_2_O_3_	Fe_2_O_3_	CaO	MgO	SO_3_	TiO_2_	K_2_O	Na_2_O	Total	Loss
Metakaolin	51.40	36.17	0.51	0.05	0.64	-	0.13	0.15	0.13	89.18	10.37
Mineral powder	33.00	13.91	0.82	39.11	10.04	-	0.10	1.91	-	98.89	1.12
Fly ash	54.29	22.55	8.46	5.58	2.56	0.53	-	1.80	0.67	96.44	2.37
Cement	21.18	4.73	3.41	62.49	2.53	2.83	-	-	0.56	97.73	1.76

**Table 2:** Fundamentally characteristics of M1, M2, and M3.

Initiation System	n(TPEG):n(AA):n(TGA)	Flowability/mm			Molecular Weight	Conversion Rate/%
		Initial	1 h	2 h		
hydrogen peroxide + ammonium persulfate-sodium bisulfite	1:2:0.1	305	280	245	55 152	67.6835
hydrogen peroxide + ammonium persulfate-Vc	1:2:0.1	340	310	290	46 178	76.6860
hydrogen peroxide + ammonium persulfate-rongalit	1:2:0.1	285	255	230	77 283	64.6282

**Table 3:** Surface tension of admixtures.

Admixture	n(TPEG):n(AA):n(TGA)	Solid Content/%	Surface Tension/(mN/m)
Pure water	-	-	72.9
M1	1:2:0.1	25%	40.12
M2	1:2:0.1	25%	42.33
M3	1:2:0.1	25%	46.40

**Table 4:** Factorial design factors and levels.

Level	Factors			
	A: n(TGA)/mol	B: n(H_2_O_2_)/mol	C: n(AA):n(TPEG)	D: n(H_2_O_2_):n(APS):n(Vc)
1	0.01	0.03	1:1	10:1:1
2	0.015	0.04	2:1	15:1:1
3	0.02	0.05	3:1	20:1:1

**Table 5:** Factorial design results.

Exp. No.	n(TGA)/mol	n(H_2_O_2_)/mol	n(AA):n(TPEG)	n(H_2_O_2_):n(APS):n(Vc)	Absorption *Q_em_* (mg·g^−1^)	Surface Tension (40%wt)/(nN·m^−1^)	Comprehensive Indices
1	1	1	1	1	1.00 (52.6)	52.7 (40.7)	56.1
2	1	2	2	2	1.19 (100)	58.6 (100)	100
3	1	3	3	3	1.15 (89.5)	45.9 (87.6)	51.1
4	2	1	2	3	1.06 (68.4)	47.6 (75.8)	46.4
5	2	2	3	1	1.11 (78.9)	44.1 (0)	39.5
6	2	3	1	2	1.11 (78.9)	57.9 (4.8)	87.2
7	3	1	3	2	0.82 (10.5)	47.4 (77.2)	16.7
8	3	2	1	3	0.76 (0)	47.8 (25.7)	12.9
9	3	3	2	1	0.88 (27.9)	50.2 (57.9)	36.2
K1	69.07	39.73	52.07	43.93			
K2	57.7	50.8	60.87	67.97			
K3	21.93	58.17	35.77	36.8			
R	47.14	18.44	25.1	31.17			

**Table 6:** The dispersivity of M4.

Sample	Dosage (%)	Saturated Absorption *Q_e_*	Surface Tension (nN·m^−1^)	Flowability (mm)		
				Initial	1 h	2 h
M4	0.15	1.34 (mg·g^−1^)	40.31	340	320	305

## Data Availability

The data is unavailable due to privacy.
